# The oceanic concordance of phylogeography and biogeography: a case study in *Notochthamalus*

**DOI:** 10.1002/ece3.2205

**Published:** 2016-06-07

**Authors:** Christine Ewers‐Saucedo, James M. Pringle, Hector H. Sepúlveda, James E. Byers, Sergio A. Navarrete, John P. Wares

**Affiliations:** ^1^ College of Biological SciencesUniversity of CaliforniaDavisCalifornia95616; ^2^ Institute for the Study of Earth, Ocean, and SpaceUniversity of New HampshireDurhamNew Hampshire03824; ^3^ Departamento de GeofisicaUniversidad de ConcepciónConcepciónChile; ^4^ Odum School of EcologyUniversity of GeorgiaAthensGeorgia30602; ^5^ Estación Costera de Investigaciones Marinas Las Cruces and Center for Marine ConservationPontificia Universidad Católica de ChileCasilla 114DSantiagoChile; ^6^ Department of GeneticsUniversity of GeorgiaAthensGeorgia30602

**Keywords:** Biogeography, Chile, connectivity, Pacific Ocean, population genetics

## Abstract

Dispersal and adaptation are the two primary mechanisms that set the range distributions for a population or species. As such, understanding how these mechanisms interact in marine organisms in particular – with capacity for long‐range dispersal and a poor understanding of what selective environments species are responding to – can provide useful insights for the exploration of biogeographic patterns. Previously, the barnacle *Notochthamalus scabrosus* has revealed two evolutionarily distinct lineages with a joint distribution that suggests an association with one of the two major biogeographic boundaries (~30°S) along the coast of Chile. However, spatial and genomic sampling of this system has been limited until now. We hypothesized that given the strong oceanographic and environmental shifts associated with the other major biogeographic boundary (~42°S) for Chilean coastal invertebrates, the southern mitochondrial lineage would dominate or go to fixation in locations further to the south. We also evaluated nuclear polymorphism data from 130 single nucleotide polymorphisms to evaluate the concordance of the signal from the nuclear genome with that of the mitochondrial sample. Through the application of standard population genetic approaches along with a Lagrangian ocean connectivity model, we describe the codistribution of these lineages through a simultaneous evaluation of coastal lineage frequencies, an approximation of larval behavior, and current‐driven dispersal. Our results show that this pattern could not persist without the two lineages having distinct environmental optima. We suggest that a more thorough integration of larval dynamics, explicit dispersal models, and near‐shore environmental analysis can explain much of the coastal biogeography of Chile.

## Introduction

Classically, the fact that marine taxa live in an “open” environment with few apparent barriers to dispersal has been presented as a paradox (Palumbi 1992): “How does diversity arise in the apparent absence of allopatry?” At the same time, there are advantages to studying the distribution of marine diversity – both the ranges of taxa and the genetic diversity within these taxa. First, in some instances, the problem can be simplified by examining diversity along a single (coastal) dimension (Gaines et al. 2009). Second, these explorations have provided new insights into how the movements of propagules in the ocean drive the diversity patterns.

For example, the physical processes of transport at Point Conception, near Santa Barbara, California, include strong coastal currents and eddies that appear to limit the northward dispersal of marine larvae across this geographic feature (Wares et al. 2001; Hohenlohe 2004). Diverging or convergent currents can have similar effects, separating coastal diversity to either side (Rocha‐Olivares and Vetter 1999; Gaylord and Gaines 2000; Hare et al. 2005); even shifts in the upwelling regime along a coast appear to have significant consequences for intraspecific as well as biogeographic patterns (Saarman et al. 2010; Barshis et al. 2011; Haye et al. 2014). Finally, species ranges themselves are influenced by the strength and the timing of physical forcing along a coast (Byers and Pringle 2006; Pappalardo et al. 2015).

At the same time, there are changes in coastal environmental conditions that may or may not be coupled with physical oceanic influences on larval dispersal. For example, complex patterns may develop when distinct coastal environments promote the divergence of populations. Hellberg (1998) showed that the range overlap among recently diverged lineages of the gastropod *Tegula* followed isolation between wave‐exposed and wave‐sheltered environments, a pattern that is also found in some tropical eastern Pacific barnacles (Meyers et al. 2013). Abundant evidence shows that “ecological” speciation or divergence is possible in marine systems (Sanford et al. 2003; Schmidt et al. 2008), so the interaction between dispersal and fitness is an important component for describing the origins of marine biodiversity.

While many idiosyncratic patterns of diversity within individual taxa exist, there has been growing recognition that the mechanisms governing transitions – both between biogeographic provinces and the genetic diversity within a single species – apply in comparable ways, so that some concordance is expected between the two types of patterns (Wares et al. 2001). To this end, a number of studies have shown a strong concordance between intraspecific and biogeographic boundaries (Dawson 2001; Wares 2002; Pelc et al. 2009; Altman et al. 2013; Haye et al. 2014).

In attempting to quantitatively describe these mechanisms of dispersal and limitation, recent work has connected empirical population genetic data with nearshore oceanographic models to explore likely causes of isolation or divergence (Galindo et al. 2006, 2010; Selkoe et al. 2010; White et al. 2010; Riginos and Liggins 2013). In general, this inferential approach has tended to focus on small spatial regions where populations harbor a significant genetic structure (Galindo et al. 2006; Nolasco et al. 2013; Sunday et al. 2014), although some explore most of a species’ range (Taylor and Hellberg 2003; Wares and Cunningham 2005; Cowen et al. 2006; Sanchez et al. 2011). The relationship between population adaptation and the mechanisms that maintain biogeographic transitions can best be understood by thorough analysis of genetic data in sister lineages that span such transitions (Sanchez et al. 2011; Dawson 2012).

**Figure 1:**
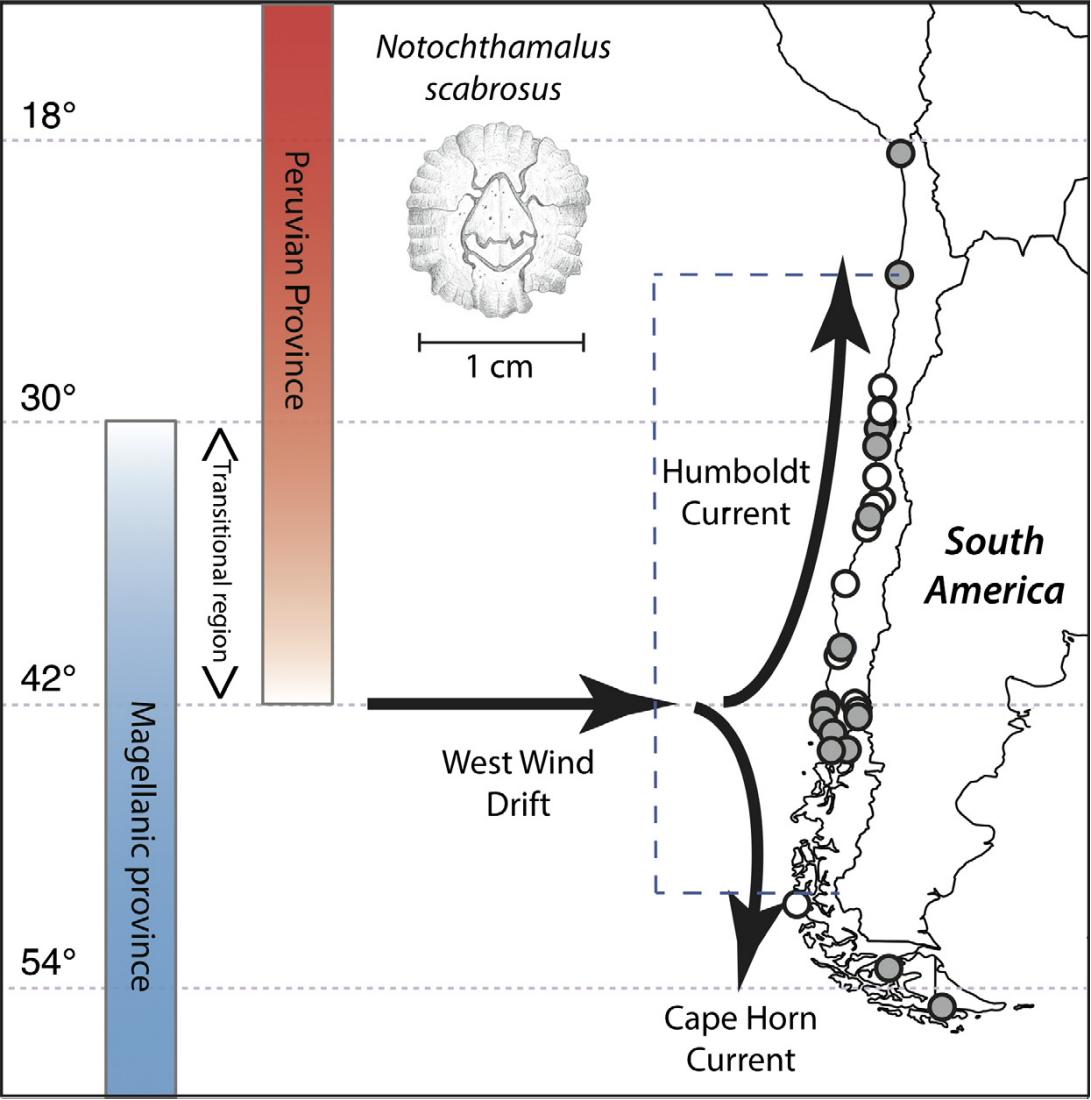
Sampling locations for *Notochthamalus scabrosus* (inset illustration Merrill 2014) along coast of South America (location details in Table 1). Mitochondrial sequence data collected at all locations; nuclear SNP data only at locations filled in gray. Major biogeographic regions on map, along with major circulation per Acha et al. (2004). Dashed box indicates domain of physical oceanographic model.

Previous work in the chthamalid barnacle *Notochthamalus scabrosus* (Darwin 1854) has shown that this intertidal species harbors high levels of intraspecific genetic diversity that appear to be coincident in structure with at least one major biogeographic transition (Zakas et al. 2009; Laughlin et al. 2012) [ZL hereafter]. Along the coast of Chile, there are two primary marine biogeographic provinces (defined by taxonomic endemicity) that are considered to broadly overlap – the “Peruvian” Province in the north, with associated taxa found as far south as ~42°S, and the “Magellanic” Province in the south (Fig. 1), with associated taxa found as far north as ~30–32°S (Brattström and Johanssen 1983; Fernández et al. 2000; Camus 2001; Thiel et al. 2007). These biogeographic transitions include not only compositional changes (range endpoints), but also major changes in abundance, recruitment, and functional structure of the rocky shore communities (Broitman et al. 2001; Navarrete et al. 2005; Wieters et al. 2009), indicating that biogeography is driven at least in part by these dynamic aspects of benthic populations. Related to the ~30°S transition, ZL identified two divergent mitochondrial lineages (using cytochrome oxidase I [COI] sequences; *d*_A_ = 0.034 ± 0.009) in *N. scabrosus*, a northern lineage found throughout the domain of the ZL studies (between 18°–40°S) and a southern lineage found only south of 30°S.

Understanding the maintenance of these related distributions entails the integration of larval ecology, physical transport, and the potential for lineages to be adapted to distinct environments across this domain. We have shown previously (Pringle and Wares 2007; Wares and Pringle 2008) that coastal transport can dominate the retention and composition of genetic diversity at a location. In this respect, oceanographic forcing is a relatively deterministic process when compared to the diffusion process modeled by stepping‐stone dispersal and many models of genetic cline distribution. Similarly, environmental factors may drive the distribution of marine lineages that are comprised of coadapted multilocus gene complexes (Schopf and Murphy 1973; Rolan‐Alvarez et al. 1997; Riginos and Cunningham 2005). We expect that these deterministic contemporary processes shape the patterns of biodiversity more than historical instances of “transient allopatry” in taxa with the broad potential for larval dispersal (Wares 2002; Sotka et al. 2004).

Here, we evaluate these interactions and the distributions of evolutionarily distinct lineages of *N. scabrosus*, using this broadly distributed taxon with high dispersal potential as a means of connecting the processes that maintain the diversity patterns along coastal Chile. By evaluating the empirical distribution of sister lineages across most of the geographic range of *N. scabrosus,* along with realistic hydrodynamics of the ocean, and the potential for differential survival or fecundity between the two lineages across this domain, we ask whether these sister distributions can be generated by larval dispersal and connectivity patterns alone, or whether spatially varying fitness at some point in their ontogeny must be invoked.

We hypothesized, given the well‐documented biogeographic transitions along the Chilean coast and the concordance of one end of the *N. scabrosus* cline with the northern end of the transition zone, that the southern lineage of *N. scabrosus* would be dominant or fixed in populations south of ~42°S latitude. At this point in the coast is a significant divergence in strong shelf currents (Acha et al. 2004) that exhibits strong shifts in salinity and water temperature, and where we expect to generate dispersal patterns that will isolate populations further to the south. Indeed, some sites in central Chile where both lineages are found exhibit high variances in lineage frequency suggestive of “sink” dynamics (Laughlin et al. 2012) seeded from populations upstream in the northward‐flowing Humboldt Current System. By sampling most of the range of *N. scabrosus*, along with far better sampling of genomic diversity using a set of anonymous SNP markers (Zakas et al. 2014), we will evaluate both the complete distribution of the mitochondrial cline and the concordance of patterns generated from nuclear DNA data.

## Methods

### Study system

#### Coastal Chile

Thiel et al. (2007) and Montecinos et al. (2012) describe the main features of coastal Chile (see also Strub et al. 1998; Fernández et al. 2000): a relatively straight, north–south coastline, with enough mesoscale geographic features, and variation in wind fields to generate a significant heterogeneity in environmental conditions and coastal circulation, each of which influences the coastal biota. At the broadest scale, the coast is heavily influenced by the Humboldt Current System from about 42°S northward (Thiel et al. 2007), and the southward‐flowing Cape Horn Current from about 42°S toward the pole (Fernández et al. 2000). There are regional patterns of upwelling strength and persistence that can have even greater influence on biological diversity, abundance, and interactions along this coast (Strub et al. 1998; Navarrete et al. 2005). In particular, much of the intertidal community and biogeographic transition between ~30–32°S is attributable to the influence of upwelling persistence on propagule recruitment to the north of this transitional region (Navarrete et al. 2005).

#### Chthamalid barnacles

Barnacles in the family Chthamalidae are globally abundant in intertidal marine habitats, generally in the highest intertidal zone (O'Riordan et al. 2010), where terrestrial environment and ecology is relevant as well (O'Riordan et al. 2004; Helmuth et al. 2006; Garcia et al. 2011; Lamb et al. 2014). Chthamalid species play a historic role in the development of competition theory (Connell 1961) and the taxon harbors a great deal of cryptic diversity (Wares et al. 2009; Govindarajan et al. 2015).

Chthamalid barnacles are all sessile and hermaphroditic. Reproduction is via copulation, followed by maternal brooding of embryos for 2–3 weeks (Hines 1978; Burrows et al. 1992), and then the release of larvae, generally peaking in late spring and summer (Hines 1978; Achituv 1986; Burrows et al. 1992; O'Riordan et al. 2004; Yan et al. 2006), although the cycle is often more complex. Larval release appears to be tied to tidal cycles, perhaps to promote larval retention (Kasten and Flores 2013). After six naupliar molts, the final cyprid larva searches for suitable habitat, attaches, and molts into a juvenile.

In laboratory trials, the pelagic larval phase of chthamalids is 15–30 days at 20°C and is longer at lower temperatures (Barker 1976; Anderson 1994; Burrows et al. 1999; Yan and Chan 2001). Evidence from field surveys suggests that the duration of the pelagic phase in the wild could be much longer (Southward 1976). Larvae are passively transported by oceanic circulation, although they can behaviorally mediate this transport via vertical swimming (Queiroga and Blanton 2005). It is thought that most barnacle larvae do not have diel migration (Tapia et al. 2010; Morgan 2014), but regulate depth during maturation. What we can observe is they are often most dense offshore at 5–10 m depth (Tapia and Pineda 2007; Tapia and Navarrete 2010; Tapia et al. 2010; Morgan 2014), with later developmental stages at depths closer to 20 m (Pfeiffer‐Herbert et al. 2007; Tapia et al. 2010). There is, of course, tremendous larval mortality (Tapia and Pineda 2007).

#### *Notochthamalus scabrosus* (Darwin 1854)

Studies of the focal species indicate that reproductive and larval development is similar to other chthamalids and dependent on water temperature (Venegas et al. 2000). Additionally, the development time from cyprid until settlement and metamorphosis is ~10 days (Venegas et al. 2000). Recruitment is greatest from October to December along the north‐central coast of Chile (Lagos et al. 2005; Tapia and Navarrete 2010), with almost no recruitment outside of this time window.

Specimens of *N. scabrosus* were collected from sites ranging from Arica, Chile (18.5°S), to Ushuaia, Argentina (54.1°S), almost the entire species range (Häussermann and Försterra 2009), with a particular focus on the biogeographic transition zones around 30° and 42°S latitude (Fig. 1, Table 1, Ewers‐Saucedo et al. 2015). Individual barnacles were identified as *N. scabrosus* in the field and immediately stored in 95% ethanol for preservation. Isolation of DNA from these tissues proceeded as in Laughlin et al. (2012). These samples were used for sequencing or haplogroup identification of mitochondrial COI and genotyping of 130 anonymous nuclear single nucleotide polymorphisms (SNP).

**Table 1:** Sampling locations with latitude, longitude, and the number of individuals for which SNP or COI data are available. SNP data were collected by BeadXpress (nuclear SNP array). COI data previously collected are available in GenBank (JQ950750–JQ951089 and GU125776–GU12595). New COI data are collected via either restriction enzyme digest with *Spe*I or sequencing (see “Methods”). New sequences are deposited in GenBank under KJ866211–KJ866385. For SNP data, average number of alleles (*N*_a_) and expected heterozygosity (*H*_e_) across loci are shown. For more information, see Ewers‐Saucedo et al. (2015)

Location	Latitude	Longitude	Nuclear SNP array *N* = (avg. *N*_a_, avg. *H*_e_)	Restriction digest/sequences of mtCOI *N* =
Arica	−18.49	−70.33	38 (1.88, 0.33)	0/28
Antofagasta	−23.65	−70.4	15 (1.76, 0.32)	0/26
Huasco	−28.46	−71.22	0	0/23
Temblador	−29.4	−71	0	0/92
Arrayan	−29.48	−70.484367	0	0/6
La Pampilla	−29.9	−71.3493691	0	0/8
Guanaqueros	−30.2	−71.43	15 (1.78, 0.33)	0/53
Punta Talca	−30.95	−71.6852778	15 (1.74, 0.32)	24/51
Los Molles	−32.25	−71.515	0	0/24
Punta Curaumilla	−33.21	−70.28	0	46/0
ECIM	−33.5	−71.62	0	76/0
Las Cruces	−33.5	−71.62	96 (1.89, 0.34)	0/61
Matanzas	−33.95	−71.85	0	46/0
Pichilemu	−34.42	−72	0	0/50
Concepcion	−36.79	−73.05	0	0/25
Valdivia	−39.8139	−73.2458	0	0/36
Niebla	−39.85	−73.4	30 (1.78, 0.32)	15/13
Apagado	−41.88	−72.58	0	48/17
Cocotue	−41.92	−73.99	35 (1.83, 0.27)	22/15
Chepu	−42.05	−74.03	38 (1.78, 0.32)	23/0
Liliuapi	−42.34	−72.44	0	24/24
Huinay	−42.35	−72.43	25 (1.64, 0.24)	21/0
Cucao	−42.63	−74.12	25 (1.69, 0.26)	41/15
Quelllon	−43.14	−73.66	24 (1.61, 0.23)	24/16
Anihue	−43.85	−72.96	24 (1.61, 0.22)	22/19
Melinka	−43.89	−73.74	16 (1.55, 0.22)	17/0
Isla Madre de Dios	−50.4251	−75.43224	0	0/11
Punta Arenas	−53.15	−70.92	39 (1.73, 0.24)	0/23
Ushuaia, Argentina	−54.8	−68.3	0	18/27

### Population genomic data

We sequenced COI as in Laughlin et al. (2012). Individuals were assigned to the northern or southern lineage using phylogenetic approaches generated with previously collected data [ZL]. Evaluation of synapomorphies separating the two primary clades led to the development of an *Spe*I restriction assay to diagnose individuals to correct clade with ~99% accuracy (as in Wares and Castañeda 2005); this was used in processing specimens from the Chiloé region and confirmed through sequencing a subsample of new individuals (Ewers‐Saucedo et al. 2015).

Genotyping of SNPs proceeded with an Illumina GoldenGate array, along with the assessment of genotypic error rate as detailed in Zakas et al. (2014). These SNPs were originally developed from specimens collected in north‐central Chile, and selected from a large number of potential loci because of ease in specific oligo development, a mix of criteria related to the classification of gene region, and all were polymorphic in our original sample from northern Chile (Zakas et al. 2014). Critically, this last criterion is a form of ascertainment bias in our SNP data: All loci are polymorphic in individuals that are considered “northern,” and all loci are biallelic (because of array genotyping); therefore, no SNPs analyzed here are diagnostic for the two lineages of *N. scabrosus*.

Single nucleotide polymorphisms were explored for outlier behavior relative to neutral expectations for a given global allele frequency using BayeScan (Foll and Gaggiotti 2008) under default analysis. Given the island‐model assumptions of this inference, our goal is not suggesting candidate loci associated with environmental transition but merely recognizing the loci that contribute the most to the overall signal. Instead, we focus on identifying loci that exhibit extreme differentiation as noted above; our threshold was for loci that exhibited a 0.99 probability of non‐neutral evolution. In addition, we evaluate cytonuclear disequilibrium between SNPs and the individual mitotype using CNDd (Asmussen and Basten 1994). Bonferroni‐corrected significance tests of association using the exact test between allele and mitotype, as well as genotype‐by‐mitotype interactions, were performed. This test was run on all populations for which the northern and southern mitotypes are sympatric, as well as a focused analysis on coastal populations between 40–42.5°S latitude where the transition of genetic lineages is most pronounced (see “Results”). Subsequent analyses were repeated, including all data and excluding the loci that exhibit outlier or disequilibrium behaviors.

#### Population differentiation

The SNP data were analyzed to generate hypotheses regarding geographic structure. Loci with >50% missing data across all individuals were excluded from the analysis. Individuals missing data from more than 25% of remaining loci were excluded as well. First, to test for the number of genetically identifiable groups, data were analyzed with STRUCTURE (Pritchard et al. 2000) for up to *k* = 5 genetic populations. We know from ZL that *k* = 1 can be rejected; *k* = 2, 3 are of relevance to the biogeographic structure of coastal Chile, and hypotheses of larger numbers of populations (*k* > 3) could suggest isolation‐by‐distance or as‐yet unrecognized population structure across the larger domain sampled in this study. Each population structure value (*k*) was explored with five replicate random‐seed analyses with 25,000 steps for statistical burn‐in and 250,000 steps for inference using an admixture model without location as prior. This approach was repeated under a no‐admixture model for contrast. Results were used to establish the most informative value of *k* using the delta‐*k* method (Evanno et al. 2005) as implemented in STRUCTUREHARVESTER (Earl and Vonholdt 2012).

Genetically divergent clusters can also be identified using discriminant analysis of principal components (DAPC; Jombart et al. 2010). In contrast to the approach using STRUCTURE, no population genetic model (e.g., Hardy–Weinberg, admixture) is assumed by DAPC, as implemented in the R package adegenet (Jombart 2008). Here, missing data were replaced with the mean frequencies of corresponding alleles, as suggested in Jombart et al. (2010). The optimal number of clusters (*k*) was chosen using Ward's clustering method based on BIC summary statistics (the “diffNgroup” selection criterion). Conclusions about population structure were weighted toward the values of *k* obtained by both analytical approaches. To evaluate the sensitivity of individual assignment to one of *k* clusters, the DAPC results are used.

Traditional pairwise *G*_st_ calculations were made under the infinite‐sites model using GenAlEx v6.5 (Peakall and Smouse 2006) with 1000 data‐by‐location permutations for statistical significance. Given prior information on *Notochthamalus* [ZL], and the range of collection sites, pairwise *G*_st_ values were calculated across all individuals as well as genotypic data partitioned by mitotype (northern or southern). The correspondence between mitotype and inferred nuclear genome identity is strongly correlated (see “Results”). These partitions of “northern” and “southern” diversity were again evaluated for pairwise site differentiation; these values of within‐lineage *G*_st_ were used to test for isolation by distance using the implementation of Mantel test in GenAlEx.

In addition to these approaches, signal of hybridization among lineages was assessed using NewHybrids (Anderson and Thompson 2002) with 5000 burn‐in iterations and 100,000 MCMC replicates to determine the Bayesian posterior probability that an individual represents one or another parental stock or hybrid class including F1, F2, or backcross. As sites of lineage sympatry generally harbor intermediate frequencies of both lineages, a uniform prior (no declared parental stock individuals) was used for mixing probabilities as in Wares et al. (2004). This approach, relying on expected transitions in allele and genotype frequencies, does not rely on diagnostic markers nor reference genotype classes.

### Physical dispersal and fitness model

#### Regional‐scale climatological ocean model

The numerical ocean model we use is an adaptation of the Regional Oceanic Modeling System (ROMS), AGRIF version (Shchepetkin and McWilliams 2005; Penven et al. 2006) described in Aguirre et al. (2012), and validated through satellite and in situ data. The model has a horizontal resolution of 1/20 degrees (~5 km), 32 sigma levels in the vertical, and a domain that extends from 23.5°S to 47°S and 70–79.5°W. The boundary conditions are based on Marchesiello et al. (2001), using a 1/2 degree (ca. 50 km) sponge layer along the open boundaries. The model is forced on the surface by climatological wind stress and heat fluxes from COADS (da Silva et al. 1994b), and the oceanic forcing was derived from WOA (Locarnini et al. 2006), with a spin‐up period of 3 years. The bathymetry is based on ETOPO2 (Smith and Sandwell 1997). The output fields were averaged and saved every 10 h. Although the model does not account for interannual or intraseasonal variability in forcing conditions, it still generates substantial interannual variability driven by internal eddy dynamics. Therefore, virtual drifters (representing barnacle larvae) were run for eight separate years, with releases in the first 5 km from shore, from all grid points, every 3 days from October through November, to represent the typical reproductive and recruitment barnacle seasons described for the Chilean coast (Navarrete et al. 2008).

#### Lagrangian particle tracking

Particles were tracked off‐line with the ARIANE package (Blanke and Raynaud 1997). We found that the statistics of dispersal were stationary when averaged over eight annual seasons. As little is known of larval depth‐related behavior, particularly in early larval stages, Lagrangian particles were released at every coastal gridpoint at 1, 10, and 20 m depths in separate experiments. From 8 years of drifter tracks, a connectivity matrix was created for every ocean grid cell adjacent to the coast. A particle was assumed to be competent to settle after 23–30 days had passed from release (Venegas et al. 2000; Navarrete et al. 2002) if found within 15 km of the coast. Larvae further from shore after the completion of the pelagic phase were assumed to die. We are constrained to consider any larvae within 15 km of the coast to have settled because finite‐difference numerical models such as ROMS only resolve features on the scale of 3–5 grid points; thus, anything with three gridpoints (about 15 km) of the coast is within the coastal boundary layer (Drake et al. 2011).

#### Connectivity probabilities and fitness modeling

The connectivity matrix defined by the eight seasons of Lagrangian particle tracks was used to drive a model for the distribution of northern‐ and southern‐type *N. scabrosus* (see Appendix 1). Barnacles can only settle when empty space is available; there is density‐dependent population growth regulated by habitat competition upon settlement (Roughgarden et al. 1988). In the model, the population growth rate is large enough that nearly all habitat is saturated; in this limit, the results do not depend on the population growth rate (Pringle and Wares 2007).

The relative fitness of the two lineages is a function of net fecundity (i.e., mortality, lifespan, remain constant) and is a step function of latitude assuming dominance of lineage type, in the respective extremes of the domain. The difference between lineages is Δ*w* (see Appendix 1). Relative fitness is assumed homogeneous within a single model grid cell, in agreement with empirical results comparing different tidal heights (Shinen and Navarrete 2014; Zakas et al. 2014). It is further assumed that habitat availability is homogeneous throughout the domain.

The model is run until the distribution of northern and southern lineages reaches a steady state, and a cost function is calculated as the squared difference in relative frequency between the model predictions and observed lineage frequencies. At each latitude, Powell's method (Press et al. 2007) is used to find the optimal Δ*w* that minimizes the mismatch between observation and data. The model is run for two sets of frequencies: the frequency at a location of the northern mitochondrial lineage and the frequency at a location of the individuals inferred to be northern based on SNP data. In the latter case, the mean assignment probability of individuals at that location is counted and used to represent the frequency of northern diversity.

## Results

**Figure 2:**
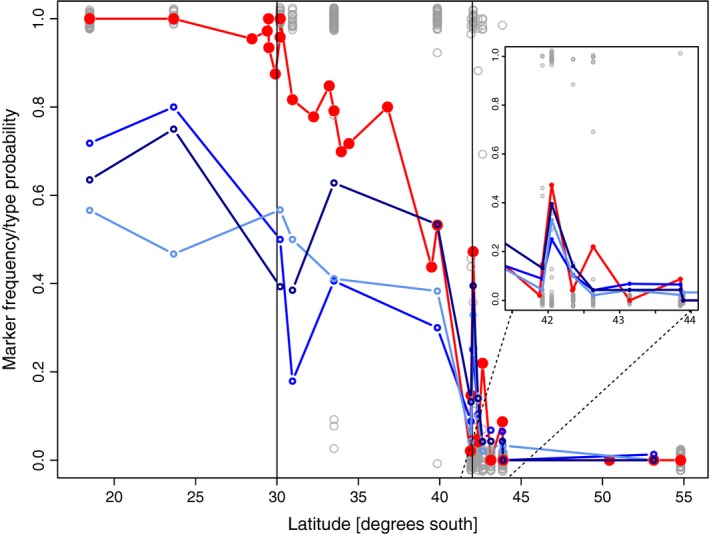
Genetic cline from latitudinal samples. Three types of data are shown. First, the red dots/lines indicate the frequency of the northern mitochondrial lineage at each sampling location. Gray circles indicate the assignment probability (vertically jittered to represent all data) to the northern lineage based on 130 nuclear SNPs (DAPC results). Individuals representing both lineages (given SNP data) can be found between 33° and 45°S; at most locations, the individuals are clearly assigned to one lineage or another, but 10 individuals between 40‐45°S have <0.9 probability of assignment to either lineage. Blue plots represent the 3 loci (see “Results”) exhibiting both a significant outlier behavior and a significant cytonuclear disequilibrium. The subset plot is shown for the Chiloé region where sample locations are closely spaced. Vertical lines delimit the region between 30° and 42°S, approximating the biogeographic transition zone shown in Figure 1. DAPC, discriminant analysis of principal components.

As reported, mtCOI sequence or mitotype data are available (Table 1, Ewers‐Saucedo et al. 2015) for 1042 individuals from 28 locations ranging from Arica, Chile (18.5°S), to Ushuaia, Argentina (54.8°S). COI sequence data from previous studies [ZL] are available in GenBank under accession numbers JQ950750–JQ951089 and GU125776–GU125954. The 174 COI sequences generated in the present study are deposited in GenBank under KJ866211–KJ866385. All pieces of information on mitotype, along with SNP data, are published at Dryad (doi:10.5061/dryad.22bc3, Ewers‐Saucedo et al. 2015). Our results show that the mitochondrial cline is effectively bounded at the north near 30°S latitude and in the south with the rapid transition from modest frequency of southern lineage at ~40°S to fixation at ~43°S (Fig. 2). Nuclear SNP data of sufficient quality were retained for *n* = 431 individuals at 130 loci; of these, 119 loci were variable (Ewers‐Saucedo et al. 2015).

### Population genomic assessment

All nuclear SNP data were evaluated for statistical behavior consistent with selection. Outlier analysis using BayeScan identified 19 loci with extraordinary statistical structure (i.e., significantly high or low *G*_st_) relative to the assumptions based on neutrality and allele frequency. Of these, eight exhibited significantly low *G*_st_ values (<0.025 in all cases). Of the remaining “high” *G*_st_ markers (*n* = 11), the range of *G*_st_ values across all sites was 0.228–0.458.

Analysis of cytonuclear disequilibrium indicated 18 SNP loci in significant (corrected for multiple comparisons) allelic disequilibrium with mitotype. Ten markers also exhibited partial genotype‐by‐mitotype disequilibrium. This analysis only included mixed locations from ~40–43°S to identify the markers in statistical disequilibrium with mitotype in a geographic region of potentially active introgression. To conservatively identify loci that have the potential to be associated with distinct environments, the overlap of these two outcomes was considered. Only three SNP markers (see Fig. 2) exhibit both high‐*G*_st_ outlier behavior (consistent with non‐neutral evolution) and cytonuclear disequilibrium (consistent with allelic interactions with the mitochondrial genome). In addition, these SNPs were within the top 15% of alleles contributing to the genetic divergence, as assessed during the DAPC analyses.

Analysis of SNP data using both STRUCTURE and DAPC indicated a most informative number of populations at *k* = 2. In STRUCTURE, the assignment probabilities to lineage were highly correlated (*r* = 0.987) regardless of whether “outlier” loci were included or not. Additionally, the analysis of STRUCTURE results using STRUCTUREHARVESTER (Earl and Vonholdt 2012) showed that *k* = 2 is returned as the best model regardless of whether the admixture or no‐admixture model is applied. DAPC shows that only 10 of the 431 genotyped individuals have a low probability (<0.9) of being assigned to either of the two lineages, indicating very clear genetic structure. Evaluation of larger numbers (*k*) of groups results in more individuals being assigned with low probability to one of the clusters. Examination of both inferences shows that north of 30°S, individuals are unmistakably assigned to the northern lineage; south of ~45°S, individuals are assigned to the southern lineage.

**Figure 3:**
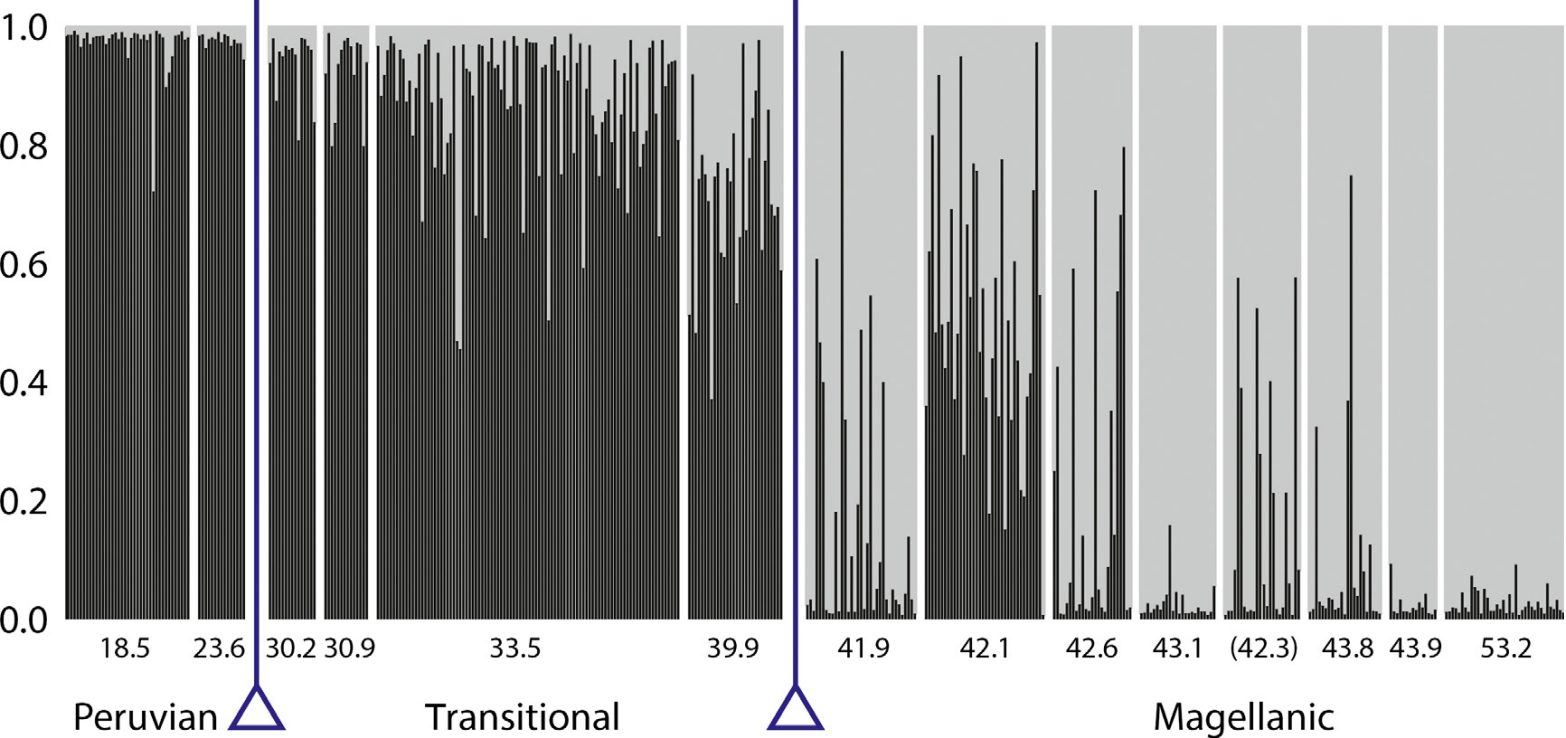
STRUCTURE results when all data are included at all locations sampled, for *k* = 2 evolutionary populations. Here, locations are represented in degrees south latitude along the Chilean coast; the site in parentheses is in the Gulf of Ancud rather than outer coast as other locations. The dark bars represent the probability of an individual belonging to the northern lineage of *N. scabrosus*, and gray represents the probability the individual belongs to the southern lineage. In this plot, the admixture model in STRUCTURE was used, leading to more uncertainty in assignment than in the DAPC results (Fig. 2) or other models under STRUCTURE analysis. Separation of biogeographic regions with triangles is provided for reference. DAPC, discriminant analysis of principal components.

Between 30–45°S, the populations are composed of individuals assigned to the northern or southern lineage at inversely related proportions across latitude, with some individuals having intermediate assignment probabilities (Fig. 2). These results are consistent between DAPC and STRUCTURE analysis (Fig. 3); the results are also the same whether using all loci, excluding the loci that are outliers or in strong cytonuclear disequilibrium, or only those loci (JPW, results not shown). Overall, the relationship between mitotype (northern or southern lineage) and assignment to nuclear group is tightly linked; binomial regression of mitochondrial lineage on assignment probability for SNP data across all data is significant (*P* < 2 × 10^−16^).

**Figure 4:**
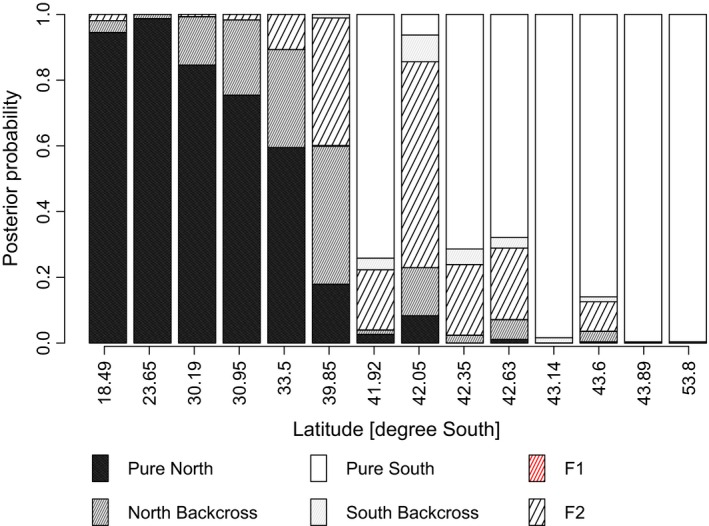
Posterior probabilities of individuals being assigned to pure northern or southern lineage, or one of the introgressed genetic classes (F1, F2, backcrosses) using NewHybrids analysis, averaged by sample location (indicated by latitude). Note that inferred F1 individuals are rare or missing (see text). The apparent imbalance toward introgression onto the northern background may be an artifact of the SNP array, which was designed for diversity of the northern distribution of *Notochthamalus*.

Evaluation of two‐generation hybrid probabilities using NewHybrids is shown in Figure 4, including only individuals from Arica (18.5°), the four outer‐coast cline region locations (Niebla, Cocotue, Chepu, and Cucao), and Punta Arenas/Ushuaia. When all individuals are included, the overall assignment to northern and southern lineages is nearly identical to this more restricted data set. The average assigned probability of an individual being an F1 hybrid is 0.0003; only one individual exhibited a moderate (>10%) probability of F1 genotype (and only in the broad‐scale analysis, not in the more geographically limited analysis). However, direct sequencing of this individual at the (diagnostic) EF1‐alpha locus as in Zakas et al. (2009) refuted this inference.

On a pairwise basis, most populations are statistically differentiated from all others given the power of multilocus SNP data, with pairwise *G*_st_ as high as 0.315 (*P* < 0.001) between Antofagasta and Añihue (~2250 km). The mean pairwise *G*_st_ among sites within the Peruvian region (north of 30°S) is 0.080, with slightly higher mean pairwise differentiation in the transitional region (locations between 30–42°S; mean *G*_st_ 0.102) and Magellanic region (south of 42°S; mean *G*_st_ 0.124). The data overall exhibit a pattern of isolation by distance; at the full scale of the study, the correlation between pairwise *G*_st_ and distance (km) is high (*r* = 0.64; *P* < 0.001), but of course includes the gradient in distribution of the two differentiated clades, which is not an indication of this equilibrium model (Wright 1943; Wares and Cunningham 2005). To avoid a tautological definition of “type” with respect to the SNP data, individuals were grouped by their mitotype and reanalyzed for isolation by distance (see above for the statistical relationship of mitotype and lineage). Under this treatment, both the northern (*r* = 0.68, *P* = 0.004) and southern (*r* = 0.46, *P* = 0.041) lineages appear to also exhibit isolation by distance. Given this understanding, it is important to note that (1) our “outlier” loci likely represent some false positives given the deviation of these data from a standard island model (Meirmans 2012); (2) any inferred support of population structure for *k* > 2 likely represents the influence of this equilibrium pattern, further supporting our focus on the two primary lineages. As we only use the outlier loci to assess the robustness of our population structure estimates, we do not repeat the evaluation of loci for non‐neutral behavior.

### Physical dispersal and fitness

**Figure 5:**
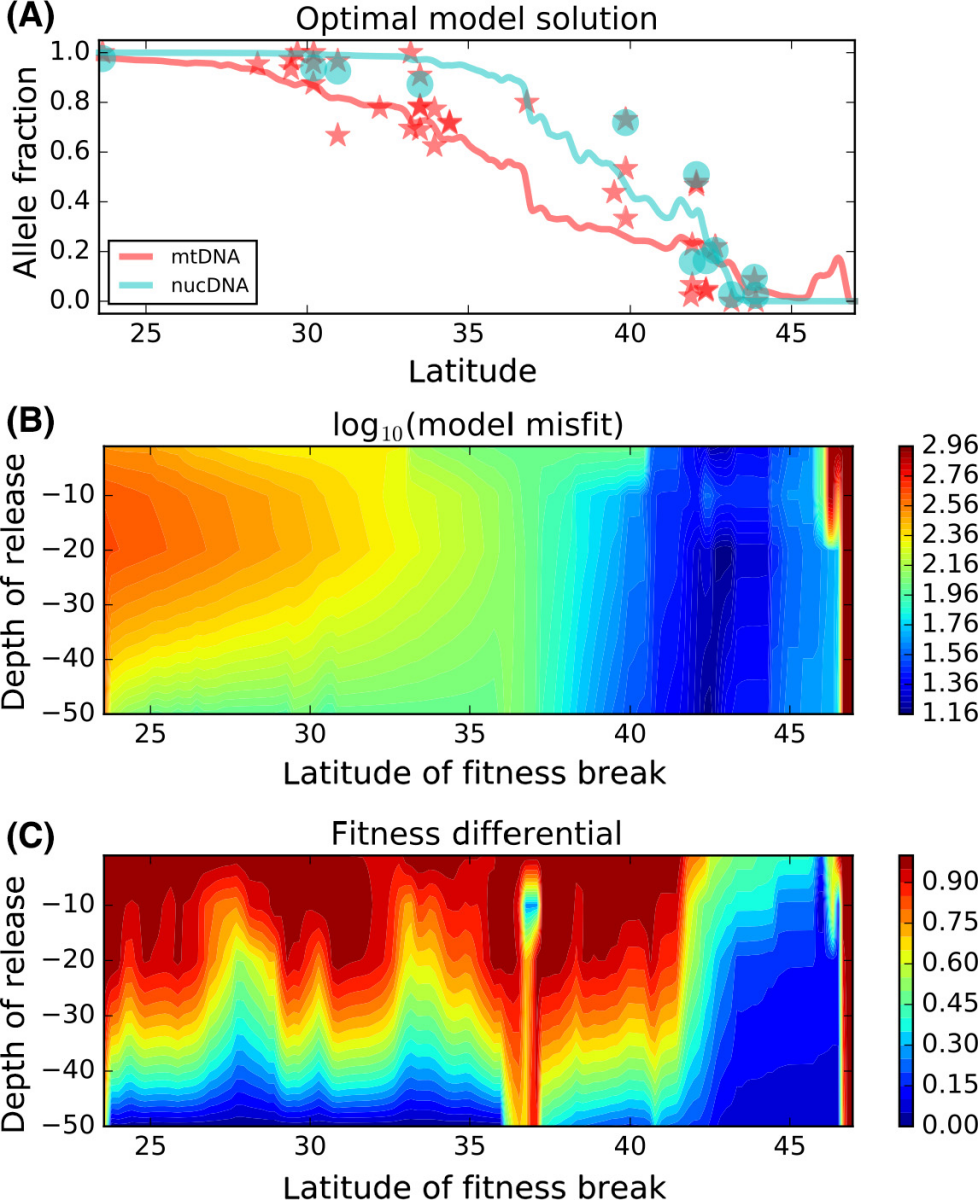
A. The northern mitochondrial lineage frequency with the lowest model misfit solution for larval release at 20 m (red line) and the observed haplotype frequencies (red stars). Some locations are sampled in multiple years and each year shown as example of interannual variation. The cyan circles and line represent the same observations and inference for the nuclear genome. B. Model misfit for the optimal fitness differential, plotted by depth of release (nearly identical for mitochondrial and nuclear observations). C. Fitness differential **Δ** *w* (Hartl and Clark 2007) that leads to lowest model misfit as function of depth of release and latitude of fitness change (nearly identical for mitochondrial and nuclear observations). Fitness is defined in terms of fecundity of locally favored allele (*R*), with fecundity of the other allele as R*(1 − **Δ** *w*). See Appendix 1 for more details.

Larval simulations were conducted at multiple release depths; at deeper releases, the mean downstream transport of larvae generally decreased, reducing the fitness differential needed to maintain the observed cline (Fig. 5). Here, we focus on particles released at 10–20 m depth, consistent with larval studies of this and related species (Millán‐Núñez et al. 1996; Millan‐Nunez et al. 1998; Vargas et al. 2006; Pfeiffer‐Herbert et al. 2007; Tapia et al. 2010); other depths are shown to indicate the robustness of our results to the exact depth of release.

The fit between the cline generated by the hydrodynamic dispersal model and empirical data is good when the change in lineage fitness occurs between 40–45°S (Fig. 5A), and the lowest cost (best fit) is found for a break between 42–43°S, depending on the depth of release (Fig. 5B). The optimal fitness coefficients are generally lowest where the cost function is lowest (better fit), and to the south of that point (Fig. 5C). As expected, the selection needed to maintain the observed spatial structure is smallest, and the model fit is best, when the fitness break is just to the south of the biogeographic transition zone at ~42°S shown in Figure 1.

The optimal fitness differential solution is unstable for fitness breaks in the region around 36°S because the model begins to be unable to find any “good” solution for fitness transitions there or northward. At that point, there are two equivalently poor solutions: zero fitness differential and equal concentrations of both lineages everywhere, or very strong fitness differential to allow the northern type to persist in the north of the domain (Fig. 5C). That both solutions are equally bad can be seen in the constancy of the model misfit across this instability (Fig. 5B).

**Figure 6:**
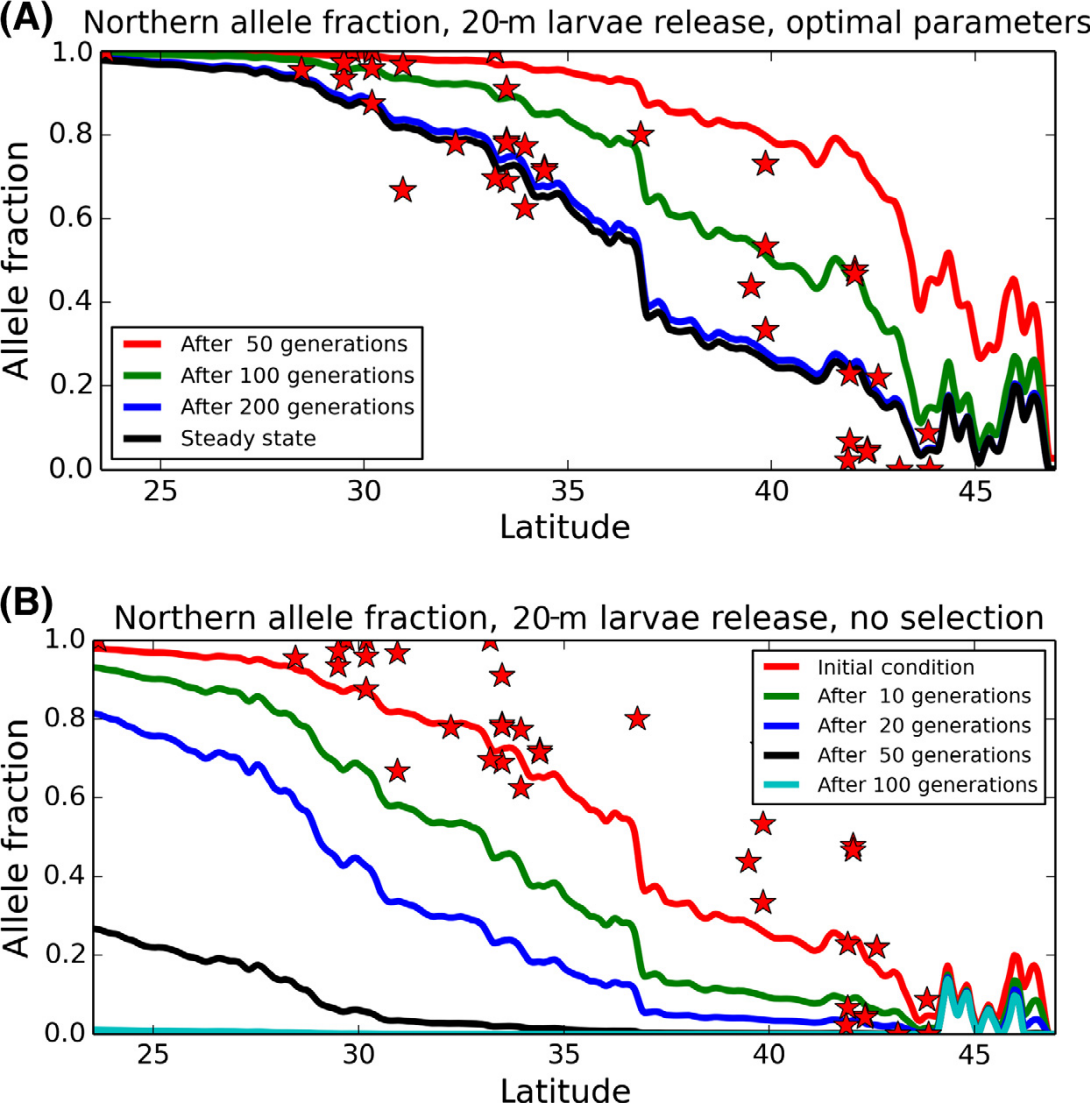
(A) The evolution of the northern‐lineage fraction from an initial condition in which the haplotype frequency is 50% at all locations. The location of the fitness break and the strength of the fitness difference are the optimal solution from Figure 5. (B) The evolution of the northern‐lineage fraction from an initial distribution based on empirical results, but with no fitness difference, through time.

To illustrate the timescale over which this cline can evolve, and its dependence on a fitness differential, non‐steady‐state model simulations were created. In model runs conducted with the optimal fitness differential and break location for 20‐m‐depth larval release, and with an initial condition of equal fractions of each mitotype, the optimal steady solution emerges in about 200 generations (Fig. 6A). If instead there is no fitness differential and the initial distribution of allele frequency matches the observed distribution, the northern allele is lost from the system in less than 100 generations (Fig. 6B). As in Wares and Pringle (2008), under neutral conditions the diversity is driven by the allele frequencies and dynamics of the upstream portion of the domain, and thus, the outcome is driven by the starting conditions. If shallower larval release depths are used, the cline will dissipate more rapidly (as evidenced by the greater fitness differential needed to maintain the cline for shallower larval releases).

## Discussion

There is a strong correlation between the pattern of overlap – and the limits to this overlap – of two evolutionarily distinct lineages in *N. scabrosus*, and the broad‐scale biogeography of coastal Chile. Although hardly indicative of a causal relationship, nor assuming a sufficient precision to analyze more extensively, we see that there is a correlation (*r* = 0.79) between the latitudinal change in frequency of genetic lineage in *N. scabrosus* and the number of biogeographic indicators of transition at each latitude along coastal Chile (Fernández et al. 2000; Camus 2001). It is important to remember that the distinction between biogeography (the patterns of described species) and phylogeography or population genetics at the same scale may be blurry; if two lineages do not diverge phenotypically, we only recover the divergence using molecular markers, and even more recent patterns may arise through the same forcing mechanisms (Dawson 2001; Wares 2002; Small and Wares 2010; Altman et al. 2013; Haye et al. 2014).

In the absence of some form of differential adaptation of the two lineages to distinct coastal environments, this diversity pattern could not persist (Fig. 6A and B). Under our model fitting approach, we are incorporating some components of relative fitness change as well as the potential for larval dynamics to influence the dispersal potential of the individuals released from a given point along the coast. Integrating our understanding of ocean physics, population genetics, and larval ecology has the potential to improve our understanding of all three components (Queiroga and Blanton 2005; Queiroga et al. 2005; White et al. 2010; Nolasco et al. 2013). For example, the insight that the depth at which chthamalid larvae have been observed offshore is *not* the depth at which coastal retention of larvae is maximized (e.g., deeper release points in the model, as shown in Figure 5, appear to require little or no fitness differential, compared to the depths at which larvae have been seen in prior studies) is useful for understanding life history evolution; similarly, the transitions in lineage frequency can happen over very short spans of coastline (e.g., Fig. 2), or even change considerably from year to year (Laughlin et al. 2012), suggesting the importance of local habitat and mechanisms associated with larval recruitment for maintaining the biodiversity at a site.

Analytical approaches to genetic clines between regional populations have traditionally relied on well‐developed theory that examines the equilibrium between gene flow and selection across an environmental gradient (Mallet et al. 1990; Szymura and Barton 1991; Sotka and Palumbi 2006); however as specified in Porter et al. (1997), this approach not only relies on the assumptions of symmetric dispersal (certainly violated in this system with strong coastal currents) but also requires diagnostic alleles to identify the lineages on either side of the clinal transition. Here, because of idiosyncrasies with our nuclear SNP markers (none of which are diagnostic for the two lineages of *N. scabrosus*) and our desire to understand how fitness and asymmetric gene flow interact (Pringle and Wares 2007), we instead use a modeling approach to understand the distribution of these two lineages. Only using this approach can we understand how sensitive our conclusions are to a limited knowledge of larval behavior and survival, yet still clarify that differential fitness – conceptually the same whether we are discussing distinct alleles, genotypes, lineages, or species (Vellend 2010) – is required to maintain the spatial patterns of lineage diversity.

A remaining uncertainty, because of the nature of the nuclear SNPs used in this study, is that we cannot be sure about the reproductive interaction of these two lineages. Uncertainty in assignment is as likely caused by ancestral polymorphism as introgression (Bulgin et al. 2003). Our sole inference of an individual with a modest probability of being an F1 hybrid is refuted by sequence data at a single nuclear locus (which is ~1% divergent between lineages and apparently lineage specific). The inferred asymmetry in genotypic composition could be associated with the probable northward flow of diversity in the Humboldt Current System (Pringle and Wares 2007; Wares and Pringle 2008), along with the northern‐lineage bias to our SNP assay (see “Methods” and Table 1 diversity indices), but our understanding of actual isolation is still poor. What is clear is that there are two quite distinct evolutionary lineages, with some markers exhibiting significant cytonuclear disequilibria even within a limited portion of the spatial range, and an overall highly significant association between mitotype and inferred nuclear lineage. This result is robust to considerations of the dynamics of individual loci, as we tested these patterns using all available data as well as excluding SNPs that appeared to exhibit extreme differentiation using standard island‐outlier models. In this respect, given that our polymorphism data are biased toward diversity from the northern part of the species distribution (see “Methods”); that we see strong evidence for two primary lineages that are concordant with the mitochondrial diversity identified previously [ZL]; and that the diversity within each lineage exhibits a pattern of isolation by distance and thus should tend to identify “false‐positive” outlier loci (Meirmans 2012) even from the modest genomic sample represented here (130 nuclear loci), we feel that we have captured the most important components of diversity in *N. scabrosus* simply by showing that it is effectively two divergent lineages, interacting both with the environment and with each other to set their complementary distributions.

In many respects, the transition zone of coastal Chile – both in terms of biogeography and in terms of the specific transition noted here in *Notochthamalus* – reflects a likely scenario of strong environmental transitions (including temperature and salinity; Acha et al. 2004) at the most prominent coastal biogeographic break (42°S), with “downstream” diversity dynamics associated with the influence of the Humboldt Current System. Our integrated analysis of ocean physics, larval ecology, and observed lineage frequencies (Fig. 5) identifies this region as the likely “selective” transition zone, and the population dynamics at certain intermediate locations (e.g., Punta Talca; see Laughlin et al. 2012) are suggestive of “sink” diversity with little local retention (Wares and Pringle 2008). The completion of the cline, coincident with the 30°S biogeographic transition and a dramatic shift in the oceanic upwelling regime (Navarrete et al. 2005), may itself not be associated with additional environmental adaptation in *Notochthamalus*. It is clear, however, that the overall pattern cannot exist without the two lineages exhibiting a distinct adaptation to the environments of northern and southern Chile; Vellend (2010) points to the conceptual ways in which selection on individual loci and the adaptation of lineages or species to particular environments can be considered in the same framework.

Here, it is also important to distinguish between the consideration of a cline using standard isotropic models that identify the equilibrium between dispersal and selection (Endler 1977; Szymura and Barton 1991; Sotka and Palumbi 2006) and those approaches that explicitly identify how the environment forces dispersal across the environment (Hare et al. 2005; Galindo et al. 2010). The example of *Notochthamalus* is, in this respect, an excellent case study for using population genetic data to infer how the environment shapes diversity, because a model based on isotropic dispersal would identify a relevant change in the selective environment near the center of the overlapping distribution of the two lineages, for example, somewhere around 36°S (see Figs. 2, 5). Our model shows this central solution to be an unstable solution (Fig. 5C), either requiring that both types are likely to be present in similar frequencies everywhere or requiring extremely high fitness differentials. Allowing for asymmetric dispersal across the domain, on the other hand, allows for relatively modest fitness differentials to be associated with a coastal region near 42°S that is already identified as exhibiting strong divergent currents (Acha et al. 2004), stronger shifts in temperature and salinity (Thiel et al. 2007), and numerous indicators of biogeographic transition (Brattström and Johanssen 1983; Fernández et al. 2000; Camus 2001).

With respect to the form of adaptation, we are not making a declaration about whether this is an example of “ecological” speciation or not; our data appear to be insufficient, both in terms of diagnostic loci and in terms of any measurement of specific environmental parameters to which this diversity could be associated with. Our analyses of “outlier” behavior for SNP loci, as well as the examination of cytonuclear disequilibrium, are not intended to identify candidate loci for local adaptation, as noted above. We believe far more (and more appropriate) information would be necessary to generate such expectations. However, our inference appears to be robust to the inclusion/exclusion of subsets of loci, as well as lineage assignment based on strictly genetic and strictly quantitative models. Additionally, our dispersal fitness model – similar to the modeling approach employed by Ayala et al. (2013) in exploring fitness differentials associated with a connectivity matrix – clearly shows that when the fitness differential between types is reduced or eliminated, the spatial structure in this system cannot persist (Fig. 6B). In this and other respects, our results are highly comparable to the work carried out in the barnacle *Balanus glandula* (Sotka et al. 2004), which also exhibits a strong coastal cline in the face of strong equatorward currents.

Still, it is not known whether the distribution of lineages in *Notochthamalus* is influenced by selection in the larval phase, or at or following settlement. Without such information, we can only speculate at the relationship between the true environmental drivers of this transition and the overall biogeography of Chile. Nevertheless, other intraspecific comparisons are relevant. The marine gastropod *Acanthina monodon* exhibits similar structure associated with the 30° and 42° biogeographic transitions (Sanchez et al. 2011); however, *A. monodon* does not have pelagic larvae. This region is the only known overlap in range for Humboldt and Magellanic penguins (Baker et al. 2006). There are no additional data comparisons to be made for an intertidal species that is distributed so broadly on the Chilean coast, with comparable larval life history (Zakas et al. 2009; Haye et al. 2014).

For species with planktotrophic larvae, environmental factors such as sea surface temperature and salinity, both of which transition rapidly near 42°, are likely to be primary factors in separating populations (Fenberg et al. 2014). Given that the Chilean coast involves divergent coastal currents, upwelling patterns, low‐salinity gulfs and fjords, and extensive temperature transitions, it is key that our data be considered in the context of transport models. In other “seascape genetics” papers where biologists have attempted to use ocean models in conjunction with genotypic data, they have not only identified the interaction of ocean and environment on distributions but also helped improve our understanding of how life history modifies that relationship. Taylor and Hellberg (2003), for instance, showed that behavioral and oceanographic processes interact to increase local larval retention in fish that have pelagic larval durations >3 weeks. Galindo et al. (2006) predicted the genetic structure of the coral *Acropora cervicornis* using ocean modeling data, and also pointed out the importance of additional biological/ontogenetic information for such studies. Other papers, taking similar approaches to that presented here, illuminate the necessity for some form of selection/adaptation to constrain the distribution of diversity (Galindo et al. 2010; Pringle et al. 2011; Nolasco et al. 2013). Of course, there can also be instances of simple, neutral patterns of population structure arising from currents and ocean physics alone (Sunday et al. 2014); the modeling work presented here shows that this is not the case for *Notochthamalus* along the Chilean coast (Fig. 6).

In fact, the reason these results are of such interest is the complex interaction between site diversity, variation in ocean circulation, and a changing environment, particularly along this coast (Aiken et al. 2011; Laughlin et al. 2012; Aiken and Navarrete 2014; Lamb et al. 2014; Shinen and Navarrete 2014). To the extent we can dissect the dynamics of this high intertidal barnacle community alone, general dispersal‐niche models proposed by Aiken and Navarrete (2014) can be better specified for the diversity that is distributed along the central coast of Chile. These populations likely exhibit high temporal variability in connectivity (again noting the temporal variation in haplogroup frequencies on Fig. 5) associated with ocean dynamics that are intimately linked to current and changing diversities throughout the Pacific Basin. If we are to understand how diversity is governed in any ecosystem, one of our primary challenges is identifying the mechanisms by which functionally similar diversity – certainly true of chthamalid barnacles (Wares et al. 2009) – is maintained in a system (Hutchinson 1959, 1961; Zhang et al. 2004; Laird and Schamp 2006; Shinen and Navarrete 2010, 2014).

## Data Accessibility

- DNA sequences: GenBank accessions JQ950750–JQ951089, GU125776–GU125954.
- SNPs developed from sequence data in NCBI BioProject PRJNA227359 (Zakas et al. 2014).
- Sampling locations, SNP genotypes, and Structure/Fst analysis outputs uploaded as Dryad doi:10.5061/dryad.22bc3, Ewers‐Saucedo et al. (2015).

## Conflict of Interest

None declared.

## Appendix 1 Methods for ocean model particle tracking

Particles were tracked off‐line with the ARIANE package (Blanke and Raynaud 1997). Data were saved every 10 h from the hydrodynamic model for particle tracking; comparison with hourly data showed that the statistics of the particle tracks were not sensitive to the data interval if it was 10 h or less for these model runs that do not include tides or synoptic variability in wind forcing. We found that the statistics of dispersal were stationary when averaged over 8 years of seasonal particle releases. As little is known of larval depth‐related behavior, particularly in early larval stages, Lagrangian particles were released at every coastal gridpoint at 1, 10, 20, and 50 m depths in separate experiments. Particles were released at each grid cell adjacent to the coast.

From the 8 years of Lagrangian particle tracks, a connectivity matrix was created for every ocean grid cell adjacent to the coast. A particle was assumed to be competent to settle after 23 and 30 days had passed from release. If found within 15 km of the coast within this time, it was assumed competent to settle in the nearest coastal gridpoint. The pelagic duration encompasses reported PLD from Nauplius I to metamorphosis at different rearing temperatures (Venegas et al. 2000). Larvae further from the shore after the completion of PLD were assumed to die.

These particle track data were used to define a connectivity matrix **E** such that each element

$$E_{\mathrm{ij}}=n_{ij}/N_{i},$$

where *n*_ij_ is the number of drifters that were released at grid cell *i* and settled at grid cell *j* and *N*_*i*_ is the number of drifters that were released at grid cell *i*. The resulting connectivity matrix was used in a population model of the regions with density dependence and spatially varying reproduction. The generations are nonoverlapping. For a northern population, the larval settlement vector along the coast at generation *k *+* *1, $L_{N^{k+1}}$ is related to the population abundance at generation *k*, $P_{N^{k}}$, by

$$L_{N^{k+1}}={\mathrm{ER}}_{N}P_{N^{k}},$$

where **R**_**N**_ specifies the fecundity matrix for the northern type; positive **R**_**ii**_ elements at each location *i* across the diagonal, and **R**_**ij**_ = 0 if *i ≠ j*. The evolution of the southern type is the same, although the fecundity **R**_**S**_ will be different. If the population of both the northern‐ and southern‐type larvae that reach a grid cell is less than the carrying capacity on the shore, *P*_max_, they are all assumed to survive (this assumption does not affect the model results, for it just modulates the fecundity matrix **R.** A reduced larval survival rate is exactly equivalent to a uniform multiplicative change to **R**). If the number of both the northern and southern larvae at a location *i* exceeds the carrying capacity of that grid cell, density dependence is used to limit the population to a uniform value in each cell to *P*_max_, and the relative fraction of the northern and southern types in that cell is determined by their ratio in the larvae settling arriving at that cell:

$$P_{Si}^{k+1}=\text{min}\;\;\left(L_{S},L_{Si}P_{\max}/\left(L_{Si}+L_{Ni}\right)\right)\;\text{and}\;P_{Ni}=\text{min}\;\;\left(L_{N},L_{Ni}P_{\max}/\left(L_{Si}+L_{Ni}\right)\right).$$

The fecundity **R** is chosen to be large enough that the population of both types pooled (Ps + Pn) is everywhere at *P*_max_, and differs for the northern and southern type as a function of their relative fitness at each location, that is, R is the subject of natural selection. Choosing a fecundity large enough that all local populations are everywhere saturated makes the results insensitive to the exact growth rate chosen and the fraction of larvae that settle which survive to adulthood (Pringle and Wares 2007). It also allows us to focus on a single life history parameter, fecundity, as the subject of selection. Note that for simplicity, *P*_max_ is the same everywhere and the two types are equal competitors (e.g., same size, interference), except for differences in fecundity. Note also that **E**, the dispersal matrix, is the same for both types.

We assume that the relative fitness of the northern and southern type of *N. scabrosus* is driven by the differences in selective pressures that can be measured in their net fecundity and that other life history parameters of the adult or larval stage (e.g., lifespan, larval behavior) are the same between types. The relative fitness of the northern and southern types is a step function of latitude assuming dominance of lineage type, with the northern type favored north of a particular latitude and the southern type favored south of that latitude; the difference between lineages is **Δ** *w* to avoid confusion with a standard relative fitness *w* setting the fitness of one type to 1. It is assumed that there are not different locations (or vertical intertidal zones) where one type can have different fitness within a single model grid cell (see Shinen and Navarrete 2014; Zakas et al. 2014). It is further assumed that habitat availability is homogeneous across the entire shoreline.

For each possible value of latitude, we seek the critical value of relative fitness such that the mismatch between the model described above and the frequency of mitochondrial haplotypes corresponding to either of the two divergent lineages of *N. scabrosus* is minimized. Starting with an arbitrary choice of relative fitness difference, the model is run to steady state. The time to achieve steady state is rapid, several hundreds of generations (and always <1000 generations), depending on the exact parameter choice. Steady state is defined as <0.1% change in the relative abundance of the northern and southern types per generation and a monotonic decrease in the rate of change in relative abundance at every location in the model. The steady‐state model solution is then used to calculate a goodness‐of‐fit metric, the squared difference of relative allele frequency between the model and the observed lineage frequencies. Using a standard minimization technique (Powell's method, see Press et al. 2007), the relative fitness parameter is then altered, the model is re‐run, and a new goodness‐of‐fit metric calculated, and the process is iterated until the relative fitness parameter that optimizes the fit between model and data is found for a given critical latitude.
